# Mechanical Properties and Heat Transfer Performance of Conically Corrugated Tube

**DOI:** 10.3390/ma14174902

**Published:** 2021-08-28

**Authors:** Zhiwei Wu, Caifu Qian, Gang Liu, Zhisheng Liu, Ping Sheng

**Affiliations:** 1 Institute of Mechanical and Electrical Engineering, Beijing University of Chemical Technology, Beijing 100029, China; qiancf@mail.buct.edu.cn (C.Q.); 15243071808@163.com (G.L.); 2 Shandong Meiling Chemical Equipment Co., Ltd., Zibo 255430, China; sdmljsk@163.com (Z.L.); sdmllzs@163.com (P.S.)

**Keywords:** conically corrugated tube, stress distribution, axial elastic stiffness, fatigue strength, heat transfer enhancement, numerical simulation

## Abstract

Conically corrugated tube is a new type of high-efficiency heat exchange tube. In this paper, the mechanical and heat transfer properties of conically corrugated tubes formed by the cold rolling of smooth tubes are studied through experimental measurement and numerical simulation to lay the foundations for applying the tubes in heat exchangers. The results show that while conically corrugated tube has a lower axial elastic stiffness compared with smooth tube, conically corrugated tube has a higher yield strength and ultimate strength. Unlike smooth tubes, conically corrugated tubes develop three-dimensional stresses when an axial tensile load is applied to them. In addition, the heat transfer coefficient of conically corrugated tube is 15%, 17%, and 115% higher than that of spiral grooved tube, convergent divergent tube, and smooth tube, respectively. Finally, the correlation equations of the axial stress concentration factor, stiffness equivalent coefficient, Nusselt number, and flow resistance coefficient of conically corrugated tubes are obtained for engineering application.

## 1. Introduction

Passive heat transfer enhancement technology [1,2] is the most commonly used method of enhancing heat transfer in heat exchangers. Changing the shape of the tube wall, such as using grooves and corrugations, helps to promote turbulence in the heat flow. The enhanced heat transfer of high-efficiency heat exchange (or HEHE) tubes is a hot issue in the field of heat transfer. Promthaisong et al. [3] investigated the effects of structural parameters (ripple depth and pitch ratio) and the Reynolds number on flow and heat transfer in spirally corrugated tubes using numerical simulation. The results showed that the vortex generated by fluid mixing in the spirally corrugated tube was beneficial to its heat transfer enhancement. Yang et al. [4] studied a new type of spirally corrugated tube by means of numerical simulation, and investigated the effects of structural parameters on its flow and heat transfer in the Reynolds number range of 10,000–35,000. The results showed that the heat transfer coefficient was the highest when the pitch ratio was 3.75; when the pitch ratio was 4.5, the comprehensive heat transfer performance was the best. Qian et al. [5] used numerical simulation to study the heat transfer and resistance performance of six-start spirally corrugated tubes and compared them with smooth tubes from the perspective of their synergy angle. Their research showed that the heat transfer performance of the spirally corrugated tube was better than that of the smooth tube, and that the friction coefficient also increased. In addition, the field synergy angle of the spirally corrugated tube was smaller than that of the smooth tube. Özden et al. [6] studied the heat transfer performance in corrugated tube using experimental and numerical simulation methods and compared it with spiral finned tube. It was found that the heat transfer coefficient of corrugated pipe was higher than that of smooth tube, but lower than that of spiral finned tube. Kareem et al. [7] studied the three-start spirally corrugated tubes through experiments and numerical simulation, taking water as the medium. It was found that the heat transfer efficiency of the spirally corrugated tubes increased by 2.4 to 3.7 times compared with the straight tube. Darzi et al. [8] studied the effect of nanofluid on flow and heat transfer in spiral grooved tubes using an experimental method. The results showed that the addition of nanoparticles could significantly improve the heat transfer performance of spiral grooved tubes. Using numerical simulation, Haergig et al. [9] studied the heat transfer performance of corrugated tubes with different structural parameters. It was found that sinusoidal and spiral grooved tubes had a better heat transfer performance. In addition, under high ripple, the ripple height had little effect on *Nu*, but the pressure drop increased significantly. Using numerical simulation, Li et al. [10] studied the flow and heat transfer performance of spirally corrugated tubes. The results showed that the heat transfer coefficient and flow resistance of the spirally corrugated tubes increased by 50–80% and 50–300% compared with the smooth spiral tube due to an additional rotation motion. Using both experimental and numerical simulation, Laohalertdecha et al. [11] studied the flow and heat transfer performance of corrugated tubes with R-134a as the medium. The results showed that the heat transfer coefficient and pressure drop of corrugated tubes increased by 22% and 19% compared with straight tubes, respectively. Zachá [12] compared and analyzed the flow field and temperature field of a spiral tube and a spiral grooved tube using numerical simulation. The results showed that the heat transfer coefficient and pressure drop of spiral grooved tube were 80–100% and 10–600% higher than that of spiral tube, respectively. Pethkool et al. [13] studied the heat transfer performance of a spiral grooved tube using an experimental method. Water was used as the working fluid in the experiment. The experimental results showed that the heat transfer coefficient of the spiral grooved tube was about 123% and 232% higher than that of a straight tube. Using an experimental method, Bharadwaj et al. [14] studied the heat transfer performance of a spirally grooved tube with a twisted tape insert. The results showed that the heat transfer performance of the spiral grooved tube improved more than in straight tubes due to the insertion of tape in the spiral grooved tube. Using an experimental method, Vicente et al. [15] studied the heat transfer performance of a spiral grooved tube with water and ethylene glycol as the media, proposed the dimensionless parameter named severity index (*Φ* = *h*^2^/*pd*), and obtained the relationship between the Nusselt number and severity index through a large number of experimental data. Through numerical simulation, Córcoles et al. [16] analyzed the influence of geometric parameters on flow and heat transfer in spiral grooved tubes under turbulent conditions and proposed a new dimensionless parameter ripple shape factor. The results showed that the Nusselt number and friction coefficient increased approximately linearly with an increase in the ripple shape factor. Hong et al. [17] used numerical simulation to compare and analyze the flow and heat transfer performance of converging-diverging tubes and converging-diverging tubes with twisted tapes. The results showed that the heat transfer coefficient and friction coefficient of converging-diverging tubes with twisted tapes increased by 6.3–35.7% and 175–530%, respectively. Khoshvaght-Aliabadi et al. [18] used both experimental and model methods to study the effects of cross-sectional dimensions (uniform, convergent, divergent, and hybrid cases) on the heat transfer and flow resistance of corrugated miniature heatsinks. The results showed that compared with the uniform cross-section, the convergent and divergent cross-section could significantly improve the heat transfer performance of the corrugated miniature heatsink and increase the pressure drop. In addition, the comprehensive heat transfer performance of the fully divergent model was the best. Lorenzini et al. [19] used experimental methods to study a new type of heatsink composed of corrugated fins. The results showed that the heatsink with the heat source distributed on the whole surface had a better performance than that with the heat source distributed locally. In addition, they noted that when the heat source was localized, it should be placed in the middle of the heatsink.

Due to the irregular wall shape of the HEHE tubes formed by the cold rolling of a smooth tube, one should consider that their bearing capacity may change significantly as a result. Li et al. [20] studied the effect of heat exchange tube stiffness on the axial thermal stress of the heat exchange tube using the finite element method. The results showed that a corrugated tube with reduced stiffness could reduce axial thermal stress compared with a straight tube. Wang et al. [21] analyzed and studied corrugated tubes using numerical simulation and obtained the relationship with stiffness. Qian et al. [22,23,24] studied the twisted pipe, bellows, spiral grooved pipe, and zoom pipe using experimental and numerical simulation methods, and proposed and calculated the stiffness equivalent coefficient of high-efficiency pipe. Shen et al. [25] studied the titanium tube in a condenser by using an experimental method. Finally, through fracture morphology analysis, it was found that the leakage in a titanium tube was caused by fatigue. Sajuri et al. [26] studied the mechanical strength and fatigue properties of bimetallic pipes with an experimental method. The experimental results showed that the fatigue strength of aluminum copper bimetallic tube was about half lower than that of copper alloy tube. In addition, the volume fraction of Cu in the material had a great influence on the bending properties of the tube. Hsu [27] studied the fatigue fracture of a titanium tube in shell and tube heat exchangers at a low temperature. The results showed that when the titanium tube was subjected to fluid induced vibration and an axial compression load, many intergranular cracks were produced in a circumferential direction resulting from composite stress.

Conically corrugated tube is a new type of high-efficiency heat exchange tube proposed by the author. In order to lay the foundations for the application of conically corrugated tubes in engineering, the mechanical and heat transfer properties of the tube were studied experimentally and numerically. Specifically, the axial bearing capacity and fatigue strength of conically corrugated tube were studied. Heat transfer performance was investigated numerically and compared with other HEHE tubes.

## 2. Experimental Measurements

### 2.1. Structure of the Studied Conically Corrugated Tubes

**Figure 1:**
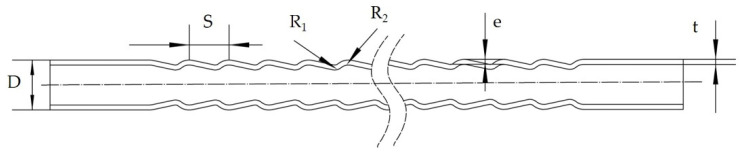
Schematic structures of a conically corrugated tube.

Figure 1 illustrates the structure of the conically corrugated tube, which is cold-rolled using smooth tube. The structural parameters *D*, *S*, *e*, *R*_1_ and *R*_2_ are the outer diameter, ripple pitch, ripple depth, trough arc radius, and crest arc radius, respectively. The material of the heat exchange tube is 1010 (No. 10 carbon steel in the Chinese steel standard). The chemical composition and mechanical properties of the material are shown in Table 1 and Table 2, respectively.

### 2.2. Axial Strength and Stiffness

**Figure 2:**
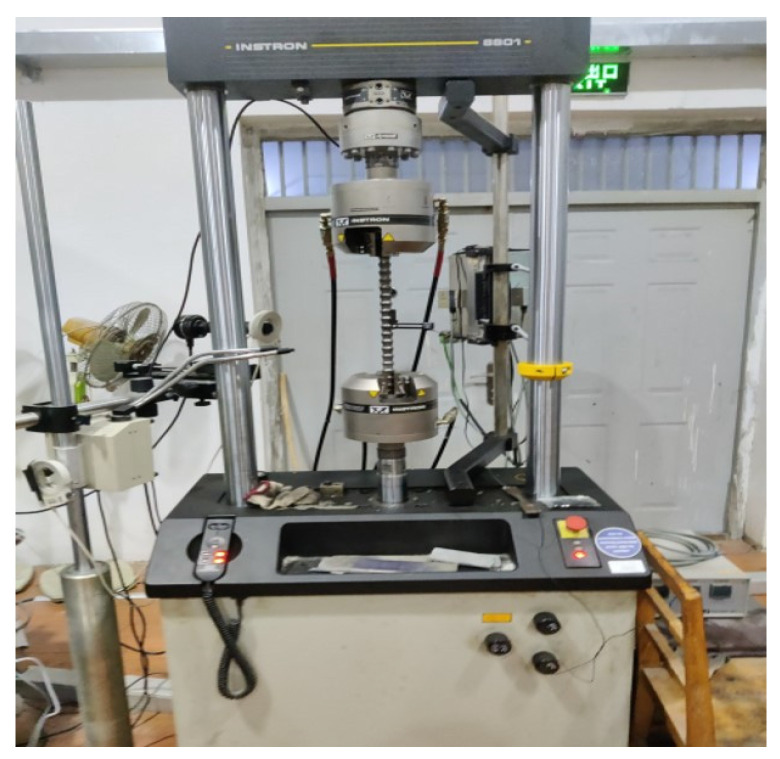
Experimental set-up.

**Figure 3:**
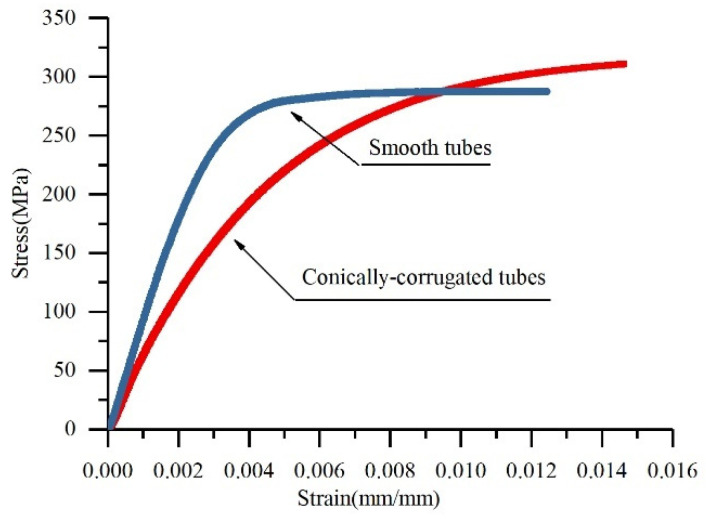
Comparison of experimental stress–strain curves of the conically corrugated tubes and smooth tubes (*D* = 25 mm, *t* = 2.5 mm).

The experiment was carried out on an Instron 8801 electro-hydraulic servo universal material testing machine (Instron Engineering Corporation, Boston, MA, USA). The range of the extensometer used in the experiment was 50 mm and the measurement accuracy was 1 micron. Figure 2 shows the experimental set-up. The axial tensile tests on conically corrugated tubes and smooth tubes were conducted by applying tensile displacement loading at the rate of 1 mm/min. The results are shown in Figure 3.

Axial stiffness [24] refers to the axial deformation of the tube under the action of unit axial tensile or compressive load at the linear elastic stage. So, the slope of the stress–strain curve in the online elastic stage represents the stiffness of the tubes. Figure 3 illustrates that the axial stiffness of the conically corrugated tubes was reduced compared with the smooth tubes. Several groups of tensile tests showed that the axial stiffness of the conically corrugated tubes was 38–43% that of the smooth tubes, and the axial stiffness of the conically corrugated tubes was about 57–62% that of the smooth tubes. However, the yield strength and ultimate strength of conically corrugated tubes may be higher than that of smooth tubes due to strain strengthening during cold-rolling.

### 2.3. Fatigue Strength

Due to changes in pressure and/or temperature, some heat exchangers are engineered to bear fatigue load. To evaluate the influence of the surface discontinuities of the conically corrugated tubes on the fatigue strength of the tubes, fatigue tests were also performed. The maximum load *F_max_* for the fatigue tests is determined as follows [24,30]:

(1) $$\sigma_{\max}=\frac{\sigma_{s}}{n}$$

(2) $$F_{\max}=\sigma_{\max}\times S$$

where *σ_s_* is the yield strength of the material, *n* is the safety factor, and *S* is the cross-sectional area of the base tube.

**Figure 4:**
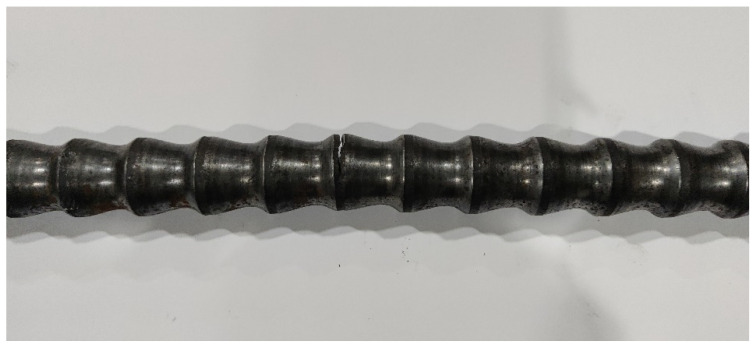
Fatigue fractured specimen.

In this study, *σ_max_* was determined by taking the safety factor as *n =* 1.5, which is the allowable load for the engineering design of heat exchanger tube. The test was carried out under tensile load. The frequency of the sinusoidal load waveform was 40 Hz and the constant load rate was 0.01. Under the same conditions, a fatigue test of the smooth tubes was also carried out. The results showed that the smooth tube did not break under 1,000,000 cycles. Table 1 lists the fatigue test results of several groups of conically corrugated tubes. Figure 4 shows the fractured specimen.

Table 3 shows that the fracture cycle of the conically corrugated tubes was at least 210,000, indicating that its fatigue strength was much lower than that of the smooth tube. Under the action of fatigue load, the working cycle of most heat exchangers is less than 100,000 times [31]. Therefore, although the fatigue strength of the conically corrugated tubes decreased, they still met the requirements for their application in engineering.

Figure 4 shows that the fracture of the specimen originated from the bulge on the surface of the conically corrugated tubes, which was caused by the stress concentration at this location. Therefore, the next section uses numerical simulations to study the stress distribution of the conically corrugated tubes under axial loading. 

## 3. Stress Analysis of the Conically Corrugated Tube

### 3.1. Numerical Models

**Figure 5:**
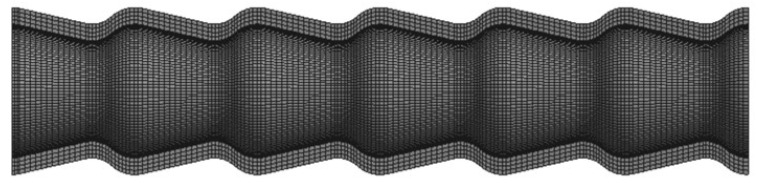
Mesh model of the conically corrugated tube.

In this study, ANSYS software (18.0) was used to study the stress distribution of the conically corrugated tubes under axial tensile load. The independence of the mesh was verified to ensure the reliability of the numerical results. The final mesh model containing 2.8 million elements is shown in Figure 5. An axial tensile load of 5000 N was applied to one end of the heat exchange tube under which the deformation of the tube was elastic. A fixed constraint was applied to the other end of the tube.

### 3.2. Stress Distribution

**Figure 6:**
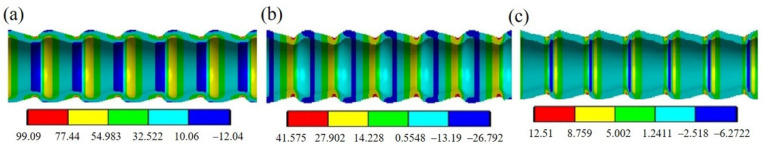
Stress distribution contours: (**a**) axial stress; (**b**) circumferential stress; (**c**) radial stress.

Figure 6 shows the three-dimensional stress distribution of the conically corrugated tube under axial tensile load. We observed, completely different from the stress state in the smooth tube, axial stress, circumferential stress, and radial stress in the conically corrugated tube. Clearly, the circumferential stress and radial stress were clearly caused by the bending moment formed by the axial load acting on the periodically changing cross-section.

**Figure 7:**
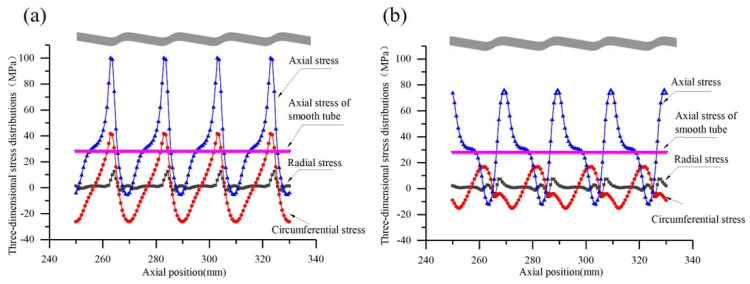
Stress distributions along tube length of conically corrugated tube: (**a**) on the outer surface; (**b**) on the inner surface (*D* = 25 mm, *S* = 20 mm, *t* = 2.5 mm, *e* = 2.5, *R*_1_ = 5, *R*_2_ = 6, *F* = 5000 N).

Figure 7 shows the axial distribution of stress on the outer and inner surfaces of the conically corrugated tubes. It can be seen that the axial stress on the inner and outer surfaces of the conically corrugated tubes was high and changed periodically. Compared with the axial stress of the smooth tube, the axial stress on the outer surface and inner surface of the conically corrugated tube increased by 2.5 and 1.7 times, respectively. In addition, although the axial stress of the conically corrugated tube varied greatly along the thickness, its average axial stress was the same as that of the smooth tube. It should be noted that the radial stress on the outer or inner surface should be understood as the stress close to the surface, because the radial stress on the surface must be zero.

Let *R_Z_^C^* be the axial stress concentration factor, defined as the ratio of the maximum axial stress in the conically corrugated tube over the axial stress in the smooth tube. It is mainly affected by the structural parameters *t* and *e*, and with sufficient simulation results, it can be fitted as a function of the dimensionless parameters ($\frac{t}{D}$ and $\frac{e}{D}$):

(3) $$\begin{array}{l} R_{Z}^{C}=-1356.023\frac{e}{D}\times {\left(\frac{t}{D}\right)}^{2}+361.25\frac{t}{D}\times \frac{e}{D}-2.287\frac{e}{D}\\ +114.392{\left(\frac{t}{D}\right)}^{2}-36.008\frac{t}{D}+3.866 \end{array}$$

The scope of application of the above formula is:

(1) *D* = 19 mm, S = 15 mm, 1.5 ≤ *t* ≤ 2.5 mm, 1.5 ≤ *e* ≤ 2.5 mm;

(2) *D* = 25 mm, S = 20 mm, 2 ≤ *t* ≤ 3 mm, 2 ≤ *e* ≤ 3 mm.

### 3.3. Axial Stiffness

The experiments revealed that the axial stiffness of the conically corrugated tube reduced to a certain extent compared with the smooth tube. Therefore, as with the stress concentration factor *R_Z_^C^*, the stiffness equivalent coefficient *K_f_^C^* is defined as the ratio of the axial stiffness of the conically corrugated tube over the axial stiffness of the conically corrugated tube. Similarly, it can be fitted to the function of dimensionless parameters ($\frac{e}{D}$, $\frac{S}{D}$ and $\frac{t}{D}$):

(4) $$\begin{array}{l} K_{f}^{C}=10.225\frac{e}{D}\times \frac{t}{D}\times \frac{S}{D}+2.996\frac{e}{D}\times \frac{S}{D}-4.397\frac{t}{D}\times \frac{S}{D}-3.686\frac{e}{D}\times \frac{t}{D}\\ -7.721\frac{e}{D}+5.195\frac{t}{D}+0.590\frac{S}{D}+0.41 \end{array}$$

The scope of application of the above formula is:

(1) *D* = 19 mm, 1.5 ≤ *t* ≤ 2.5 mm, 1.5 ≤ *e* ≤ 2.5 mm,13 ≤ *S* ≤ 19 mm.
(2) *D* = 25 mm, 2.00 ≤ *t* ≤ 3.00 mm, 2 ≤ *e* ≤ 3 mm,14 ≤ *S* ≤ 22 mm.

Equation (4) was verified by experimental measurements as listed in Table 4, where *K_f_^E^* is the stiffness equivalent factor obtained by experiments, *K_f_^C^* is the stiffness equivalent coefficient obtained by Equation (4), and the relative error is defined as *(K_f_^C^* − *K_f_^E^*)/*K_f_^E^*. Thus, the error was less than 10%, which is acceptable in engineering.

## 4. Study on Heat Transfer Performance

### 4.1. Physical Model

**Figure 8:**
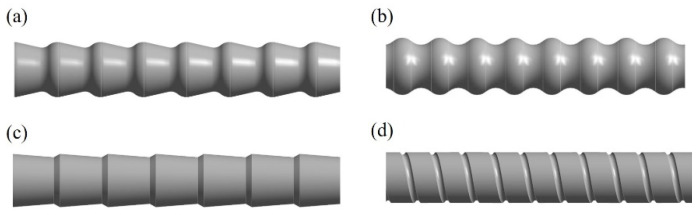
Geometric models of HEHE tubes: (**a**) conically corrugated tube; (**b**) arc-corrugated tube; (**c**) converging-diverging tube; (**d**) spiral grooved tube.

Next, the fluid flow and heat transfer performance in the conically corrugated tubes were numerically studied. For comparison, are-corrugated tubes, spiral grooved tubes, and converging-diverging tubes were also numerically simulated. Figure 8 shows the geometric models of the fluid in these tubes. It should be noted that in order to avoid the influence of the inlet and outlet section of numerical simulation on the flow and heat transfer effect in the tube, straight tube sections were provided at both the inlet and outlet of the tube.

### 4.2. Governing Equations

For steady-state conditions, the following equations are involved as continuity, momentum, and energy equations [16,18]:

(5) $$\frac{\partial \left(\rho u_{i}\right)}{\partial x}=0$$

(6) $$\frac{\partial \left(\rho u_{i}u_{j}\right)}{\partial x_{j}}=-\frac{\partial p_{i}}{\partial x_{i}}+\frac{\partial }{\partial x_{j}^{}}\left[\mu \left(\frac{\partial u_{i}}{\partial x_{j}}+\frac{\partial u_{j}}{\partial x_{i}}\right)\right]$$

(7) $$\rho \frac{\partial u_{j}T}{\partial x_{j}}=\frac{\partial }{\partial x_{j}}\left(\frac{\lambda }{c_{\rho }}\frac{\partial T}{\partial x_{j}}\right)$$

In the simulation, the fluid flow was completely turbulent. The standard *k-ε* turbulence model was used for turbulent region. The turbulent flow energy *k* and the dissipation rate ε were calculated by the following equations:

(8) $$\begin{array}{l} \rho \frac{\partial k}{\partial t}+\rho \frac{\partial \left(ku_{j}\right)}{\partial x_{i}}=\frac{\partial }{\partial x_{i}}\left[\left(\mu +\frac{\mu_{t}}{\sigma_{k}}\right)\frac{\partial k}{\partial x_{j}}\right]+G_{k}-\rho \varepsilon \end{array}$$

(9) $$\rho \frac{\partial \varepsilon }{\partial t}+\rho \frac{\partial \left(\varepsilon u_{j}\right)}{\partial x_{i}}=\frac{\partial }{\partial x_{i}}\left[\left(\mu +\frac{\mu_{t}}{\sigma_{\varepsilon }}\right)\frac{\partial \varepsilon }{\partial x_{j}}\right]+\frac{C_{1\varepsilon }\varepsilon }{k}G_{k}-C_{2\varepsilon }\rho \frac{\varepsilon^{2}}{k}$$

(10) $$G_{k}=-\rho \bar{u_{i}^{\prime }u_{j}^{\prime }}\frac{\partial u_{j}}{\partial x_{i}}$$

(11) $$\mu_{t}=\rho C_{\mu }\frac{k^{2}}{\varepsilon }$$

where *C*_1*ε*_, *C*_2*ε*_, *C_μ_*, *σ_k_*, and *σ_ε_* are all constants with the values being 1.44, 1.92, 0.09, 1.0, and 1.3, respectively.

### 4.3. Concerned Properties

The heat transfer rate *Q* is expressed by the following equation:

(12) $$Q=M\times c_{\rho }\times \left(t_{out}-t_{in}\right)$$

The heat transfer coefficient is defined as:

(13) $$h=\frac{Q}{S\times \Delta t_{m}}$$

(14) $$Nu=\frac{h\times d_{e}}{\lambda }$$

where *S* is the heat transfer area; *λ* is the thermal conductivity; *d_e_* is the characteristic diameter; Δ*t_m_* is the logarithmic average temperature difference between the fluid and the tube wall.

The local heat transfer coefficient (*h_x_*) was computed at each location as follows:

(15) $$h_{x}=\frac{q_{x}}{T_{w_{x}}-T_{f_{x}}}$$

where $q_{x}$ is the heat flux at position x in the tube; $T_{w_{x}}$ and $T_{f_{x}}$ is the average inner wall temperature and the average overall fluid temperature at position x, respectively.

The friction factor (*f*) was computed according to the Darcy equation:

(16) $$f=\Delta p\frac{2}{\rho u^{2}}\frac{d}{L}$$

where *f* is the friction coefficient, Δ*p* is the total pressure difference, *L* is the length of the pressure section, and *u* is the average fluid velocity.

### 4.4. Boundary Conditions and Numerical Approach

The boundary conditions used for fluid flow and heat transfer simulation are summarized as follows: the non-slip boundary conditions were applied to all fixed walls; the tube wall temperature was a constant temperature set at 293 K; water was selected as the working fluid and the inlet fluid temperature was set to 323 K; the scalable wall function method was adopted; a simple algorithm was used to couple the pressure field and velocity field. Moreover, the momentum equation, turbulent energy equation, and dissipation rate equation were discretized by the second-order upwind formula. The physical parameters of the water are shown in Table 5 [32].

### 4.5. Grid Generation and Independence Tests

**Figure 9:**
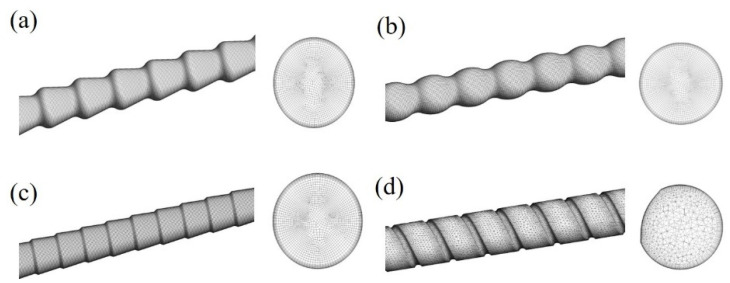
Grid models of HEHE tubes: (**a**) conically corrugated tube; (**b**) arc-corrugated tube; (**c**) converging-diverging tube; (**d**) spiral grooved tube.

The grid models of heat exchange tubes are shown in Figure 9. In order to ensure the accuracy of the numerical results, grid independence tests on all tubes were performed and the final grid models were determined.

**Figure 10:**
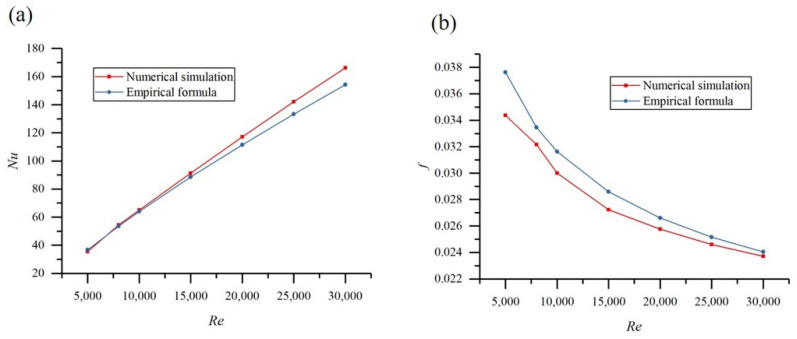
Verification of numerical simulations: (**a**) *Nu* and (**b**) *f*.

In this study, fluent software was used to simulate the fluid flow and heat transfer inside tubes. In order to verify the reliability of the simulation results, the Nu and f of the smooth tube with an inner diameter of 20 mm under 5000 ≤ *Re* ≤ 30,000 were computed and compared to those obtained by the empirical formula [32], as shown in Figure 10. It is shown that the absolute value of relative error was less than 8%, which implied that the numerical simulation was reliable.

### 4.6. Results and Discussion

**Figure 11:**
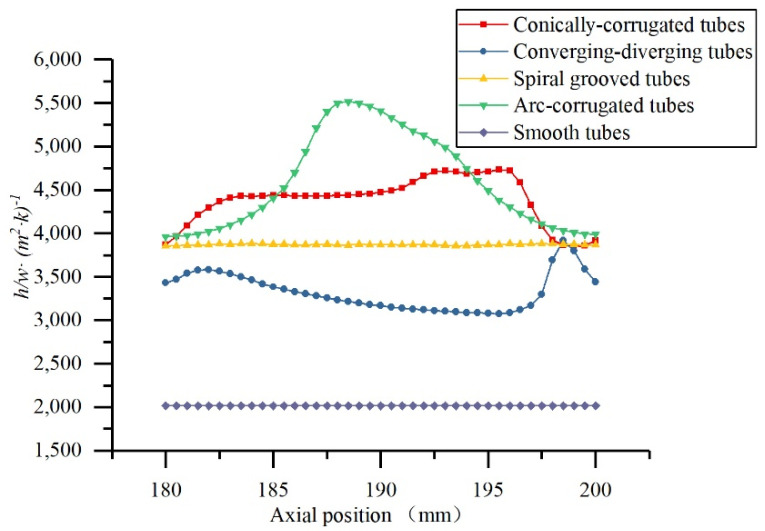
The local heat transfer coefficient plotted at each plane at Re = 10,000 for HEHE tubes.

Figure 11 shows the local heat transfer coefficient at each plane in different axial locations. It is shown that the heat transfer performance of HEHE tubes was much better than that of the smooth tube in the studied models. The heat transfer performance of the HEHE tubes was ranked from high to low as conically corrugated tube/arc-corrugated tube, converging-diverging tube, and spiral grooved tube.

### 4.7. Effects of Reynolds Number on the Heat Transfer and Flow Resistance

**Figure 12:**
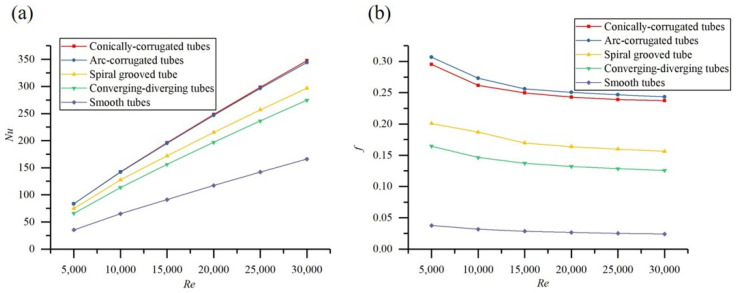
Variations in (**a**) *Nu* and (**b**) *f* with Reynolds number for HEHEs.

Figure 12a shows the variation in the Nu with Reynolds number for the studied tubes. It is clear that the Nu increased with the increase in the Reynolds number, and that all of the HEHE tubes had a better Nu than that of the smooth tubes. The Nusselt number of the conically corrugated tubes was about 15%, 17%, and 115% higher than that of the spiral groove tubes, converging-diverging tubes, and smooth tubes, respectively. Figure 12 shows the variation in the resistance coefficient with Re for the HEHE tubes. The flow resistance of all the HEHE tubes was higher than that of the smooth tube. It is worth noting that the resistance coefficient of the conically corrugated tube was better than that of the other HEHE tubes.

### 4.8. Correlation Equations for Heat Transfer and Flow Resistance

A large number of numerical simulations was carried out on the heat transfer performance and flow resistance of the conically corrugated tube by considering the effects of its geometric parameters, fluid properties, and flow state. The correlation equations for the heat transfer coefficient and flow resistance of the conically corrugated tubes were obtained using a multiple regression technique.

(17) Nu=0.236Re0.83Pr1/3μμw0.14ed0.838Sd−0.465R1d−0.052

(18) f=110.334Re−0.061(ed)2.82(Sd)−1.556(R1d)−0.307

The application scope is *d* = 20, 5000 ≤ *Re* ≤ 30,000, 0.8 ≤ $\frac{S}{d}$ ≤ 1, 0.1≤ $\frac{e}{d}$ ≤ 0.15, 0.15 ≤ $\frac{R_{1}}{d}$≤ 0.35, and 0.695 ≤ *Pr* ≤ 216.023. It should be noted that *d* is the inner diameter of the base tube.

Numerical verification showed that the deviation between the simulation results and the calculation results obtained by the correlation equations was within 10%, which meant that the correlation equations for calculating the heat transfer and flow resistance of the conically corrugated tubes are reliable and appropriate.

## 5. Conclusions

In this study, the mechanical and heat transfer properties of conically corrugated tubes were studied using experimental measurements and numerical simulations. The main conclusions are as follows:

- The experimental results showed that compared with the smooth tubes, the axial elastic stiffness of the conically corrugated tubes was lower, but the yield strength and the ultimate strength could be higher.
- Although the fatigue strength of the conically corrugated tubes was not as good as that of the smooth tube, it can still meet the requirements for engineering applications.
- Unlike the smooth tubes, axial, circumferential and radial stresses existed in the conically corrugated tube when placed under axial load, and the axial stress of the conically corrugated tube was much greater than that of the smooth tube under the same conditions.
- The axial stress concentration coefficient and stiffness equivalent coefficient of the conically corrugated tubes were defined and regressed into the function of structural parameters, which could be used in the engineering of the tube’s strength design.
- Under the studied conditions, the heat transfer coefficient of the conically corrugated tubes was about 15%, 17%, and 115% higher than that of spiral grooved tubes, converging-diverging tubes, and smooth tubes, respectively. In addition, the Nusselt number and resistance coefficients of the conically corrugated tubes were proposed and verified for its reliability and applicability.

It should be noted that this paper is only concerned with conically corrugated tubes. A more practical study should take a whole heat exchanger using conically corrugated tubes as its object and perform a comprehensive study including fluid flow, heat transfer simulations or experiments, and making a mechanical design of the equipment.

## Figures and Tables

**Table 1:** Chemical composition table of 1010 (wt %) [28].

Material	C	Si	Mn	S	P	Cr	Ni	Cu
1010	0.07–0.13	0.17–0.37	0.35–0.65	≤0.035	≤0.035	≤0.15	≤0.25	≤0.25

**Table 2:** Mechanical properties of the 1010 material (at room temperature) [29].

Tensile Strength (MPa)	Yield Strength (MPa)	Elongation (%)	Section Shrinkage (%)	Hardness (Unheated) (HBs)
≥335	≥205	≥31	≥55	≤137

**Table 3:** Fatigue experimental results of the conically corrugated tubes.

No.	Tube Size						Safety Factors	Materials	Cycles	Results
	*D*	*S*	*E*	*R1*	*R2*	*t*				
1	25.0	20.0	2.5	5.0	6.0	2.5	1.5	1010	520,000	broken
2	25.0	20.0	2.5	5.0	6.0	2.5	1.5	1010	210,000	broken
3	25.0	20.0	2.5	5.0	6.0	2.5	1.5	1010	350,000	broken
4	25.0	20.0	2.5	5.0	6.0	2.5	1.5	1010	650,000	broken

**Table 4:** Experimental verification of *K_f_^C^* for the conically corrugated tubes.

No.	Tube size						*K_f_^C^*	*K_f_^E^*	Results
	*D*	*S*	*e*	*R1*	*R2*	*t*			
1	25.0	20.0	2.5	5.0	6.0	2.5	0.6178	0.56226	−8.9%
2	25.0	20.0	2.5	5.0	6.0	2.5	0.5889	0.56226	−4.52%
3	25.0	20.0	2.5	5.0	6.0	2.5	0.5769	0.56226	−2.53%

**Table 5:** Physical properties parameter of water.

Parameter	Value
*_Cp_*	4174, J kg^−1^·K^−1^
*μ*	0.000601, kg m^−1^·s^−1^
*ρ*	990.2, kg·m^−3^
*λ*	0.642, Wm^−1^·K^−1^
